# Amebic Encephalitis in a Patient with Chronic Lymphocytic Leukemia on Ibrutinib Therapy

**DOI:** 10.1155/2018/6514604

**Published:** 2018-08-01

**Authors:** Ensi Voshtina, Huiya Huang, Renju Raj, Ehab Atallah

**Affiliations:** ^1^Department of Medicine, Medical College of Wisconsin, Milwaukee, WI, USA; ^2^Department of Pathology, Medical College of Wisconsin, Milwaukee, WI, USA; ^3^Department of Hematology and Oncology, Medical College of Wisconsin, Milwaukee, WI, USA

## Abstract

Chronic lymphocytic leukemia (CLL) is the most common type of leukemia in Western countries. A common first-line therapy offered to qualifying patients includes ibrutinib, an oral covalent inhibitor of Bruton's tyrosine kinase. Treatment of CLL with ibrutinib therapy is generally well tolerated; however, serious opportunistic infections are being reported in patients treated with ibrutinib. In this report, we present a patient with CLL on ibrutinib therapy who developed rapidly declining neurological status concerning for the central nervous system (CNS) process related to his immunocompromised status. Despite multiple testing modalities, no evidence was found to explain the acute changes the patient was experiencing, and he had no improvement with common antimicrobial coverage. The patient ultimately expired, and autopsy of the brain revealed granulomatous amebic encephalitis due to opportunistic infection by *Acanthamoeba* species. As evidenced by this case, ibrutinib therapy, despite being generally well tolerated, has the potential to predispose patients to opportunistic infections like amebic encephalitis. Amebic encephalitis is a highly lethal CNS infection, and it is important for clinicians to recognize early on the potential for infection in patients on ibrutinib therapy presenting with CNS symptoms.

## 1. Introduction

Chronic lymphocytic leukemia (CLL) is the most common type of leukemia in western countries with an estimate of about 20,000 newly diagnosed cases [1]. Clinical course of CLL patients is highly variable and partly depends on the stage of the disease. Staging is commonly performed using the Rai staging or the Binet staging [2]. An impaired immune system predisposes CLL patients to frequent infections which is a common cause of death in these patients. Treatment for CLL is chosen based on cytogenetic abnormalities, age, and performance status of the patient. Currently, the first-line therapy offered to unfit elderly patients with multiple comorbidities as well as CLL patients with 17p deletion includes ibrutinib, an oral covalent inhibitor of Bruton's tyrosine kinase [2, 3]. Ibrutinib is generally well tolerated, and the rate of infections reported in clinical trials with ibrutinib was comparable to historical controls; however with long-term follow-up, serious infections are being reported in patients treated with ibrutinib [4–7].

The most common infection in patients with CLL undergoing immunosuppressive treatment has been associated with bacteria and frequently involves the respiratory and urinary tracts [8]. Amebic encephalitis is an extremely rare and highly lethal central nervous system (CNS) infection, with mortality rate above 90% [9]. The initial symptoms can be indistinguishable from bacterial meningitis or mimic a brain abscess, bringing challenge to early diagnosis. We report a patient with CLL who developed an opportunistic infection with amebic encephalitis while on ibrutinib therapy.

## 2. Case Presentation

The patient was a 72-year-old male who presented to the emergency department (ED) with complaints of headache and seizure-like activity with shaking of his bilateral upper extremities. His past medical history was significant for CLL with 13q deletion diagnosed 6 years prior to presentation. He was treated at that time with fludarabine and rituximab for 4 cycles; however, he was not able to complete a 5th cycle due to prolonged cytopenia. Two years later due to progression of disease, he was started on ibrutinib 420 mg daily and continued for 2 years. He developed severe neutropenia while on ibrutinib, and treatment was held for two months until resolution. He presented to the ED one month after resuming ibrutinib.

**Figure 1:**
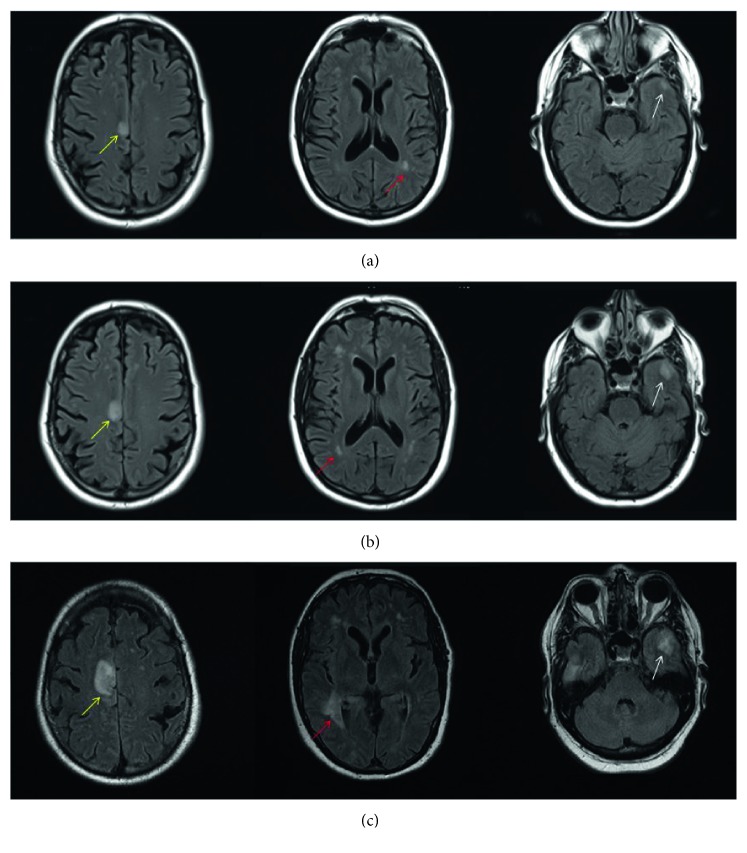
Axial T2-FLAIR MRI brain. (a) MRI at initial presentation shows the focal area of increased signal in the right frontal cortex (yellow arrow) along with areas of abnormal white matter signal abnormality in the left occipital lobe (red arrow) and the left temporal lobe (white arrow). (b) MRI obtained 2 days later with the increase in size of signal abnormality involving the right frontal cortex (yellow arrow) and multiple areas of signal abnormality in the right frontal, right occipital (red arrow), and left temporal lobes (white arrow). (c) Ten days from initial presentation, there were new and increase in previously known long TR hyperintensities involving the cortical (yellow arrow) and subcortical (red and white arrows) white matter. Given the short interval imaging findings, an infectious etiology was favored as the cause of the patient's symptoms.

At presentation, the patient was alert with the only examination finding of episodic shaking movements. He was afebrile and had a leukocytosis of 15,200/*µ*L with 66% lymphocytes. Initial workup included computed tomography (CT) of the head without contrast which showed no findings to explain presenting symptoms. Continuous electroencephalography (EEG) evaluation was negative for epileptiform activity. With persistent symptoms he was started on anticonvulsants with levetiracetam and phenytoin. A magnetic resonance imaging (MRI) of the brain with contrast was obtained, and it showed a nonspecific focal area of increased signal involving the right frontal cortex (Figure 1(a)). He was transferred to our institution for further workup and management.

On arrival, the patient was evaluated for progression of CLL by the chest, abdomen, and pelvis CT which was negative for any evidence of disease with no lymphadenopathy or splenomegaly. MRI of the spine was performed and was negative for any disease other than degenerative changes. Ophthalmological evaluation was unrevealing for any intraocular pathology. He was worked up for autoimmune processes with ANA, CRP, and ESR, all of which were insignificant. Infectious workup with blood culture, urine culture, quantiferon-TB, histoplasma, blastomyces, influenza, RPR screen, and HIV were negative. He had a lumbar puncture which showed cerebrospinal fluid (CSF) with elevated WBC 97/*µ*L, polysegmented neutrophils (PMN) 2%, lymphocytes 82%, red blood cells (RBC) 4/*µ*L, elevated protein 93 mg/dL, and normal glucose of 44 mg/dL. FilmArray meningitis and encephalitis panel was negative for all the following tested agents: *Escherichia coli*, *Haemophilus influenzae*, *Listeria monocytogenes*, *Neisseria meningitidis*, *Streptococcus* group B, *Streptococcus pneumoniae*, *Cytomegalovirus*, *Enterovirus*, *Herpes simplex* virus 1 and 2, *Human herpesvirus* 6, *Human parechovirus*, *Varicella zoster virus*, and *Cryptococcus neoformans*. Gram stain and culture also turned out to be negative. Cytologic analysis of CSF was negative for malignant cells or large cell transformation. CSF flow cytometry showed a minute population of 0.04% CD5+ B-CLL cells, which was not felt to be clinically significant and sufficient to explain the acute changes the patient was experiencing. Other CSF tests, which were negative, included angiotensin-1-converting enzyme, John Cunningham (JC) polyomavirus, cryptococcal antigen, fungal culture, and CSF toxoplasma serologies.

The patient became increasingly lethargic and started complaining of worsening headache. He also started having high fevers which persisted despite treatment with broad-spectrum antibiotics with vancomycin and piperacillin/tazobactam and antivirals with acyclovir. His neurological status continued to decline and repeat MRI brain showed new and increase in size of previously known scattered hyperintensities with associated rim enhancement (Figure 1(c)). At this time, infectious etiology was favored, given the acuity of changes seen on imaging and patient status. The infectious coverage was expanded and included IV vancomycin, cefepime, and ampicillin for bacterial meningitis; IV amphotericin B for atypical fungal meningitis; and IV acyclovir was continued for viral encephalitis. The patient continued to decompensate clinically, and a repeat CT of the head showed hydrocephalus. An external ventricular drain was placed which did not improve his status. A leptomeningeal biopsy was not performed considering the difficult location and size of the lesions. As all workup continued to be unrevealing, progressive multifocal leukoencephalopathy (PML) was thought to be the most likely diagnosis to fit the patient's medical history and imaging and laboratory results; however, JC polyomavirus was tested negative on two separate CSF analyses, making this diagnosis unlikely. The patient ultimately expired within two weeks after he presented with the neurologic symptoms after support was withdrawn per family request, and an autopsy was performed.

**Figure 2:**
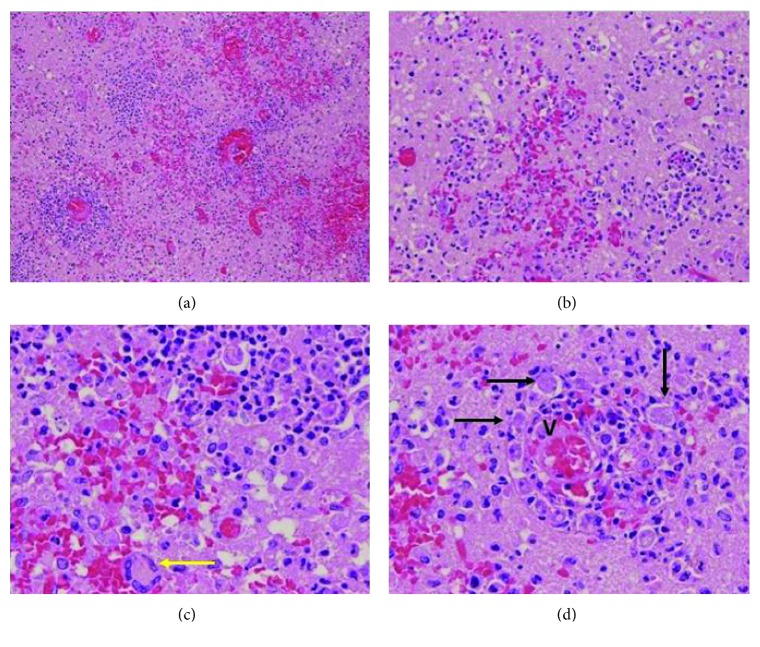
Microphotographs of autopsy brain sections. Hematoxylin and eosin stain. (a) A low-power view shows areas of parenchymal hemorrhage and necrosis with mixed inflammation, ×10 objective. (b) A higher power view demonstrates amebic trophozoites and occasional cysts, ×20 objective. (c) At high power, numerous amebic trophozoites can be seen intermixed with inflammatory cells and occasional multinucleated giant cells (yellow arrow), ×40 objective. (d) The amebae (black arrow) are prominent around vessels (V), ×40 objective.

Autopsy of the brain showed diffuse cerebral edema with right-sided predominance on gross findings. There were multiple areas of hemorrhagic necrosis including the bilateral frontal lobe, right parasagittal posterior frontal lobe, left temporal lobe, bilateral medial occipital lobe, and paraventricular areas. Microscopic findings revealed parenchymal necrosis with mixed inflammation, amebic trophozoites, and occasional cysts (Figures 2(a) and 2(b)). This mixed infiltrate was seen involving the meninges. The amebas were prominent around vessels, and occasional multinucleated giant cells were seen (Figures 2(c) and 2(d)). The autopsy brain was sent to the Centers for Disease Control and Prevention (CDC) and immunohistochemical stains were performed, which identified *Acanthamoeba* species with no evidence of *Naegleria fowleri*. His final diagnosis based on autopsy was necrotizing meningoencephalitis with morphologic and immunohistochemical evidence of *Acanthamoeba* species.

## 3. Discussion

A wide array of conditions can cause neurological symptoms in CLL patients. The incidence of leptomeningeal involvement of CLL is rare and has been reported at 0.8 to 2% based on autopsy case studies [10, 11]. Another concern is that CNS symptoms could represent a more malignant variant such as diffuse large B-cell lymphoma transformation as seen in Richter's syndrome (RS). Just as in CLL CNS involvement, only a few cases of isolated RS without systemic lymphoma have been reported [12]. Oftentimes, CLL cells tend to recruit to sites of inflammation in both infectious and inflammatory processes and to discern them as the etiology for the CNS manifestation can be clinically challenging [13]. A B-cell monoclonal lymphocytosis >5% by flow cytometry in the CSF was found to be associated with clinically significant CNS involvement with CLL [14]. In our particular patient, because of the lack of pathological evidence of large cell transformation and the small amount of CLL cells seen in the CSF, CLL was not considered to be the etiology of neurological symptoms. Moreover, he was on ibrutinib which has been shown to cross the blood-brain barrier and have CNS penetration with other forms of non-Hodgkin's lymphoma [15]. Considering all of these factors, CNS involvement by CLL was not rendered as the clinical diagnosis.

The most commonly described neurologic complication in patients with CLL is opportunistic CNS infection [16]. One such infection-associated complication is PML, which is a demyelinating process in the CNS caused by JC virus infection. HIV infection accounts for 85% of PML cases. PML is also associated with hematologic malignancies such as CLL, with a reported incidence rate of 11.1 per 100,000 person-years [17, 18]. Treatment with various chemotherapeutic agents has been linked to the development of PML [19–22]. There has been one case report of PML after treatment with ibrutinib therapy in a patient with CLL [23]. MRI often shows asymmetric lesions. Diagnosis via CSF analysis by PCR in immunosuppressed patients has been shown to have a sensitivity of 93% and specificity of 99% for detecting JC virus [24], though biopsy-proven disease remains to be the gold standard for diagnosis. In our case, however, both CSF analysis and autopsy results were negative for JC virus infection thus excluding PML.

Risk of infection and its complications in CLL patients have been associated with disease status and treatment-related immunosuppression [25, 26]. Ibrutinib therapy has been associated with a high rate of infection of 148.6/100 person-year, especially in patients receiving treatment for relapsed or refractory CLL [5]. The majority of infections are bacterial, but there have also been case reports of rare opportunistic infections with military tuberculosis, invasive aspergillosis, cryptococcal meningoencephalitis, and disseminated fusarium infection [6, 27–31]. The most commonly seen atypical infection is found to be pneumocystis jiroveci pneumonia [7].

Amebic encephalitis is a rare CNS infection with high mortality rate caused by free-living amebae [9]. There are two entities of amebic encephalitis, primary amebic meningoencephalitis (PAM) and granulomatous amebic encephalitis (GAE). PAM caused by infection with *Naegleria fowleri* is a rapidly fatal hemorrhagic encephalitis typically occurring in immunocompetent children and young adults swimming in fresh water or inadequately chlorinated pools. GAE is less common and is due to opportunistic infection in immunosuppressed or debilitated hosts by *Acanthamoeba* species, most commonly found in lakes, tap water, and heating and air conditioning units, or *Balamuthia mandrillaris* [32]. Although *Acanthamoeba* encephalitis has also been reported in immunocompetent hosts [33], it is a mostly insidious and almost uniformly fatal encephalitic process [34], with acute and fulminant cases also reported [35, 36]. The patient usually presents with fever, nonspecific neurological symptoms, and enhancing edematous brain lesions found on MRI, but enhancement may or may not be seen despite the presence of an aggressive, necrotizing, parasitic infection [29]. The low incidence, nonspecific presentation, and imaging findings lead to difficulty in early diagnosis of the infection. Treatment regimens are mostly anecdotal and not well defined as most of the cases are diagnosed on autopsy. Our patient received amphotericin B and did not respond. Despite its rarity, clinicians should remain aware of this disease especially in immunocompromised patients. To our knowledge, this is the first case of amebic encephalitis in a patient receiving ibrutinib therapy for treatment of progressed CLL.

## 4. Conclusion

In this report, we present a patient with CLL on ibrutinib therapy who developed an opportunistic infection with amebic encephalitis. This is an extremely rare and highly lethal CNS infection that currently has no standard of treatment. It is important for clinicians to recognize the potential for infection in patients receiving ibrutinib therapy.
